# Clinical characteristics and long-term outcome of patients with gastrointestinal involvement in eosinophilic granulomatosis with polyangiitis

**DOI:** 10.3389/fimmu.2022.1099722

**Published:** 2023-01-12

**Authors:** Rongli Li, Yingying Chen, Shangzhu Zhang, Linyi Peng, Jiaxin Zhou, Yunyun Fei, Wen Zhang, Yan Zhao, Xiaofeng Zeng

**Affiliations:** ^1^ Department of Rheumatology and Clinical Immunology, Peking Union Medical College Hospital, Chinese Academy of Medical Sciences, Peking Union Medical College, Beijing, China; ^2^ National Clinical Research Center for Dermatologic and Immunologic Diseases (NCRC-DID), The Ministry of Education Key Laboratory, Beijing, China

**Keywords:** eosinophilic granulomatosis with polyangiitis, gastrointestinal involvement, outcome, systemic vasculitis, Churg-Strauss syndrome

## Abstract

**Objective:**

This study aims to investigate clinical characteristics, potential risk factors, as well as long-term outcome in EGPA patients with GI involvement.

**Methods:**

A total of 94 EGPA patients were included in this cohort study. We retrospectively reviewed the clinical data, treatment, and outcome of 21 EGPA patients with GI involvement and compared them with other 73 EGPA patients without GI involvement. Multivariate logistic regression was used to find potential risk factors associated with GI involvement in EGPA patients.

**Results:**

Compared with EGPA patients without GI involvement, EGPA patients with GI involvement had higher level of hs-CRP (65.1 (24.5-138.9) vs. 21.3 (5.7-39.1) mg/L, p=0.005), higher grades of Birmingham vasculitis activity score (BVAS) (20 (13-29.5) vs. 12 (16-19), p=0.022), higher Five Factor Score (FFS) (1 (1-2) vs. 0 (0-1), p<0.001), and were more likely to have weight loss (66.7% vs. 38.4%, p=0.021) at baseline. In EGPA patients with GI involvement, the most common gastrointestinal symptoms were abdominal pain (90.5%) and diarrhea (42.9%). Weight loss was identified as a potential risk factor for GI involvement in EGPA patients (OR = 4.304, 95% CI 1.339–13.841). During follow-up, EGPA patients with GI involvement showed lower 1-year cumulative survival rate (75.2% vs. 100.0%, P <0.0001) and 3-year cumulative survival rate (67.7% vs. 100.0%, P<0.0001), lower long-term remission rate (33.3% vs. 86.3%, P<0.001), but higher 1-year cumulative relapse rate (19.2% vs. 3.8%, P=0.03) and 3-year cumulative relapse rate (54.6% vs. 13.1%, P<0.001) compared with patients without GI involvement.

**Conclusion:**

EGPA patients with GI involvement had distinct features from those without GI involvement, including higher hs-CRP level, higher BVAS and FFS scores. EGPA patients with GI involvement showed lower cumulative survival rate, lower long-term remission rate and higher cumulative relapse rate compared with those without GI involvement.

## Introduction

Eosinophilic granulomatosis with polyangiitis (EGPA) is a systemic disease characterized by severe asthma, hypereosinophilia and multiple organ involvement due to vasculitis affecting small- to medium-sized vessels (1, 2). Lungs, nose, peripheral nerves, skin, gastrointestinal (GI) tract, heart and kidneys were commonly involved in EGPA patients (1, 3–8), and typical histopathological finding revealed eosinophilic infiltrates, necrotizing vasculitis and extravascular granuloma in affected tissues (1, 6, 9, 10). The prevalence of GI involvement in EGPA patients ranged from 8% to 59% according to past studies, mainly manifesting as abdominal pain, diarrhea, nausea/vomiting, GI bleeding and perforation (7, 11, 12). Severe GI involvement could be life-threatening and was acknowledged as a poor prognostic factor in EGPA patients (13). Therefore, early recognition and immediate management are required to decrease morbidity and mortality of EGPA patients with GI involvement.

So far, there have been few large-sample studies demonstrating the clinical features of GI involvement in EGPA patients, and most studies about GI involvement in EGPA patients were case reports or small case series. Herein, we conducted a retrospective study to summarize the clinical and laboratory features, treatment, long-term outcome and potential risk factors of EGPA patients with GI involvement in a relatively large cohort.

## Patients and methods

We retrospectively reviewed all 94 hospitalized EGPA patients in Peking Union Medical College Hospital from December 2001 to August 2021. All patients fulfilled the America College of Rheumatology classification criteria of EGPA (14). Patients’ data during their first hospitalization were determined as baseline data. The appearance of initial symptoms associated with EGPA was defined as disease onset, and disease duration was defined as duration from disease onset to first hospitalization. Demographic and clinical data of EGPA patients were recorded at baseline and during follow-up, including gender, age at disease onset, disease duration, initial symptoms, clinical manifestations, organ involvement, laboratory tests, imaging findings, endoscopic evaluation, histological examination, treatment, and outcome. Laboratory tests included complete blood count, urinalysis, liver and renal function tests, erythrocyte sedimentation rate (ESR), hyper-sensitivity C-reactive protein (hs-CRP), Antineutrophil Cytoplasmic Antibodies (ANCA) test, serum immunoglobulin E (IgE) levels. As for imaging findings, results of ultrasonography (US), digital radiography (DR), barium meal radiography, computed tomography (CT), positron emission tomography/computed tomography (PET/CT), magnetic resonance imaging (MRI) were recorded if available. Results of endoscopic evaluation included gastroscopy, colonoscopy, and capsule endoscopy were also recorded if applicable. In addition, histopathological results were also recorded when patients underwent tissue biopsies. We also evaluated all patients’ Birmingham vasculitis activity score (BVAS) (15, 16) and revised Five Factor Score (FFS) (13). As an important item reflecting the severity of EGPA, we also added EGPA related weight loss at baseline as an important factor in the analysis, other causes of weight loss beside EGPA were all excluded in the study.

We further divided EGPA patients into two groups according to baseline data: patients with GI involvement and patients without GI involvement. GI involvement was defined as: (1) eosinophilic infiltration of GI mucosa or GI tract vasculitis confirmed by histopathological examination; and/or (2) GI symptoms supported by radiologic or endoscopic evidence and cannot be explained by other underlying causes other than EGPA; and/or (3) GI manifestations present at diagnosis or recurrence of vasculitis and improve after the treatment of immunomodulatory agents (17–19). We compared the clinical data, treatment, and outcome of EGPA patients with GI involvement and EGPA patients without GI involvement. During follow-up, remission was defined as the absence of clinical systemic manifestations due to vasculitis (3, 20, 21), and relapse was defined as worsening disease manifestations requiring dose increase of glucocorticoids and/or addition or switch of immunosuppressive medication (21, 22).

The study was approved by the Ethics Committee of Peking Union Medical College Hospital and was conducted in accordance with the Helsinki Declaration. Written informed consent was waived due to the retrospective nature of this study.

### Statistical analysis

Continuous variables were shown as mean ± standard deviation (SD) or median (interquartile range [IQR]), and categorical variables were shown as the number (percentage). Continuous variables were analyzed using the Student’s t-test or Mann-Whitney *U*-test, while categorical variables were analyzed using the Chi-square test or Fisher’s exact test, as needed. The Kaplan-Meier curves and Log-Rank test were used to calculate the cumulative relapse rate and survival rate. Multiple logistic regression was used to explore independent factors for GI involvement, which was presented with odds ratios (ORs) and 95% confidence intervals (CIs). All the statistical tests were completed by IBM SPSS statistics (Version 25.0, IBM, Armonk, NY, USA) and GraphPad Prism 8 (GraphPad Software, Inc., La Jolla, CA, USA) software. A *p*-value < 0.05 was defined as statistically significant difference.

## Result

### Baseline data and clinical manifestations of EGPA patients

A total of 94 EGPA patients were included in this study, including 60 (63.8%) males and 34 (36.2%) females. At baseline, 40 (42.6%) patients were untreated, 23 (24.5%) patients were receiving glucocorticoids monotherapy, and 31 (33.0%) patients were receiving a combination therapy of glucocorticoids and immunosuppressants. The mean age of EGPA onset was 47.2 ± 14.5 years and the median course of the disease was 42 months (IQR, 12-96 months). Laboratory findings revealed that all patients had blood eosinophilia (median Eosinophil count (10^9^/L) [IQR]: 4.1 [1.81-8.36]). ANCA-positivity were detected in 30 (31.9%) patients, among which MPO-ANCA and PR3-ANCA positive patients accounted for 19 (63.3%) and 2 (6.7%), respectively.

Paranasal sinus abnormality and asthma were the most common clinical manifestations, observed in 77 (81.9%) and 75 (79.8%) patients respectively. In addition, peripheral neuropathy and skin involvement occurred in 63 (67.0%) and 50 (53.2%) patients respectively. Regarding to the internal organ system, lung was the most common affected organ (88.3%), followed by heart (28.7%), GI tract (22.3%), kidney (21.3%) and central nervous system (14.9%).

### Characteristics of EGPA patients with or without GI involvement at baseline

**Figure 1:**
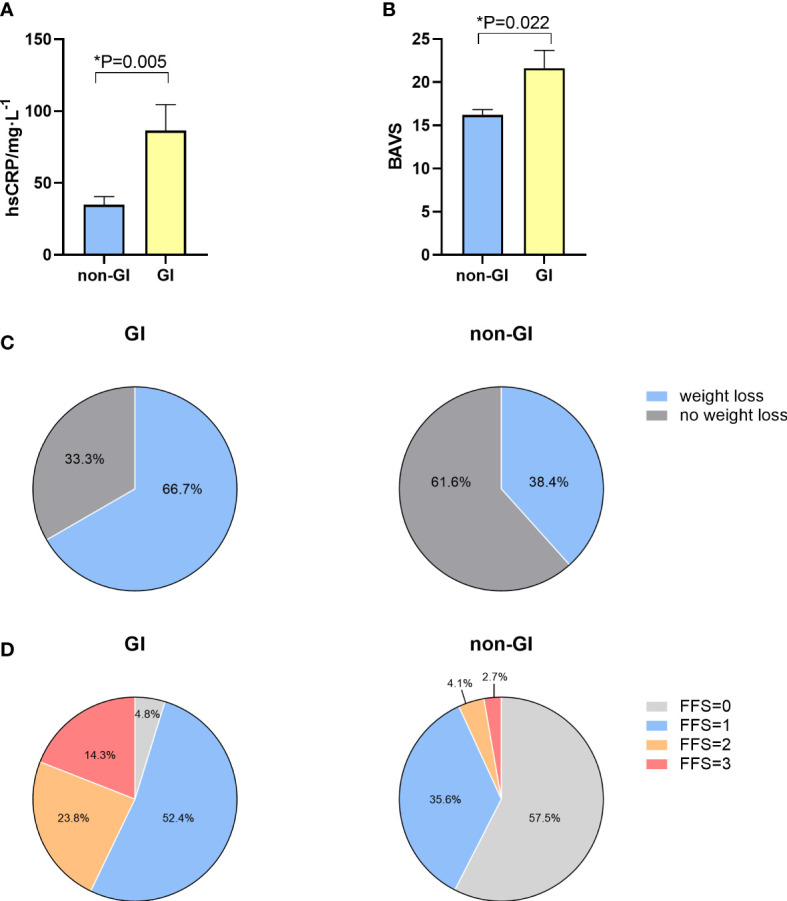
Distinct characteristics between EGPA patients with and without GI involvement.

Twenty-one (22.3%) EGPA patients had GI involvement in this cohort. We divided EGPA patients into two groups, patients with GI involvement (GI group) and patients without GI involvement (non-GI group). In contrast with non-GI group patients, GI group patients had higher serum hs-CRP level (65.1 mg/L (24.5-138.9) vs. 21.3 mg/L (5.7-39.1)), p=0.005) (**Figure 1A**) and higher grades of Birmingham vasculitis activity score (BVAS) (20 (13-29.5) vs. 16 (12-19), p=0.022) (**Figure 1B**). Moreover, compared with non-GI group patients, GI group patients were more likely to experience weight loss (66.7% versus 38.4%, p=0.021, **Figure 1C**), and higher Five Factor Score (FFS) (1 (1-2) vs. 0 (0-1), P<0.001, **Figure 1D**). However, no differences were found between EGPA patients with or without GI involvement in terms of age, organ involvement, treatment at baseline, eosinophil count, ESR or serum IgE levels. Detailed data are shown in **Table 1**.

**Table 1:** Characteristics of EGPA patients with or without GI involvement.

	GI group	Non-GI group	P value
	(n=21)	(n=73)	
Demographic features			
Gender (Female (%))	8 (38.1%)	26 (35.6%)	0.835
Age at EGPA onset (yr) Duration before diagnosis (m) Patients received GCs at baseline Baseline GCs dosage (Prednisone or equivalent dose, mg/d) Baseline GCs duration (m)	40.0 ± 16.9 36 (12-102) 15 (71.4%) 30.0 (10.0-45.0) 1.0 (0.5-3.0)	41.5 ± 14.1 48 (13-96) 39 (53.4%) 35.0 (15.0-60.0) 1.0 (0.3-1.3)	0.683 0.445 0.141 0.382 0.455
Clinical manifestations			
Weight loss	14 (66.7%)	28 (38.4%)	***0.021***
Skin involvement	12 (57.1%)	38 (52.1%)	0.680
Nasal cavity involvement	16 (76.2%)	61 (83.6%)	0.521
Asthma	16 (76.2%)	59 (80.8%)	0.758
Renal involvement	5 (23.8%)	15 (20.5%)	0.766
Cardiac involvement	6 (28.6%)	21 (28.8%)	0.986
Peripheral neuropathy	13 (61.9%)	50 (68.5%)	0.571
CNS manifestation	5 (23.8%)	9 (12.3%)	0.294
Laboratory tests			
ANCA positivity Eosinophil count (10^9^/L) Eosinophil (%) ESR (mm/h) hs-CRP (mg/L) Serum IgE (kU/L)	5 (23.8%) 3.3 (1.9-9.0) 34.8 (23.8-45.0) 30.0 (6.0-64.0) 65.1 (24.5-138.9) 332.0 (164.5-646.5)	25 (34.2%) 4.2 (1.7-8.1) 37.3 (20.2-50.9) 33.5 (16.0-57.0) 21.3 (5.7-39.1) 418.0 (164.0-1847.0)	0.433 1.000 0.865 0.703 ***0.005*** 0.383
Scoring			
BAVS	20 (13-29.5)	16 (12-19)	***0.022***
FFS			
0 1 2 3	1 (4.8%) 11 (52.4%) 5 (23.8%) 4 (19.0%)	42 (57.5%) 26 (35.6%) 3 (4.1%) 2 (2.7%)	***<0.001*** 0.166 ***0.013*** ***0.021***

when p value< 0.05, it is highlighted in bold.

### Characteristics of GI involvement in GI group patients

**Figure 2:**
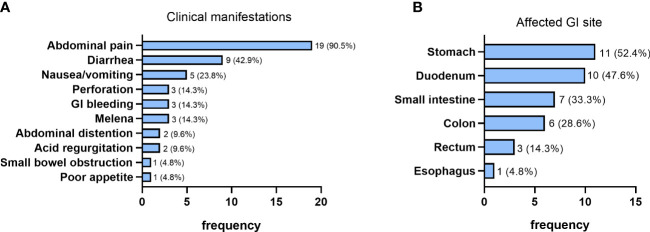
Clinical manifestations **(A)** and the affected site **(B)** of patients with GI involvement (n=21).

Of the 21 patients in GI group, only 6 (28.6%) patients had GI manifestations as their initial symptoms at EGPA onset. Abdominal pain and diarrhea were the most frequent symptoms in patients with GI involvement, occurring in 19 (90.5%) and 9 (42.9%) patients respectively (**Figure 2A**). Furthermore, nausea/vomiting, GI perforation, GI bleeding and melena were also relatively common manifestations, with a prevalence of 23.8% (5/21), 14.3% (3/21), 14.3% (3/21) and 14.3% (3/21), respectively. Abdominal distention and acid regurgitation were recorded in 2 (9.5%) patients, respectively. Small bowel obstruction and poor appetite were found in 1 (4.8%) patient each (**Figure 2A**).

18 patients (85.7%) in GI group completed gastroscopy, while the other 3 patients failed to complete gastrointestinal endoscopy due to acute GI bleeding, GI perforation and intestinal obstruction, respectively. The most common finding through gastroscopy was nonulcerative gastritis, which was found in 14 patients (77.8%). Among them, 13 patients had only chronic superficial gastritis which presented as edema, hyperaemia or erosion of gastric mucosa, and one patient had chronic atrophic gastritis. The incidence of duodenitis was also relatively high, which occurred in 7 (38.9%) patients and manifested as edema, hyperaemia or erosion of duodenal mucosa. Moreover, gastroduodenal ulceration was found in 4 (22.2%) patients, among which 2 patients had gastric ulceration and 2 patients had duodenal ulceration. Esophagitis was less common and was found in 2 (11.1%) patients.

As for lower digestive tract endoscopy, 9 patients undertook colonoscopy examination, and one patient performed capsule endoscopy simultaneously. Colitis was the most common finding observed by colonoscopy, which was found in 5 (55.6%) patients. More specifically, 3 patients had ulcerative colitis presented as multiple ulcers of colon, while 2 patients were found to have nonulcerative colitis manifesting as edema, hyperaemia or erosion of colonic mucosa. Besides, proctitis was observed in 3 (33.3%) patients, among which 1 patient had ulcerative proctitis and the other 2 had nonulcerative proctitis. However, multiple colorectal polyps occurred only in 1 (11.1%) patient. Furthermore, one patient was found to have ulcerative enteritis according to the capsule endoscopy. Histopathological results of 15 patients indicated that 15 (100%) patients with GI involvement were diagnosed with acute and/or chronic gastrointestinal inflammation, while eosinophilic infiltration was detected in 11 (73.3%) patients and granuloma formation was observed in one (6.7%) patient. Nevertheless, chronic inflammation with intestinal metaplasia and hyperplastic polyps were relatively rare, with an incidence of 13.3% (2/15) and 6.7% (1/15) respectively.

We further divided the GI group patients into two subgroups. Group I included 12 patients whose histopathological results confirmed the involvement of GI tract. The other 9 patients were taken into Group II, which included 7 patients who did not undergo GI tract biopsy due to extremely severe GI involvement (6, 85.7%) and hernia (1, 16.7%) and 2 patients whose histopathological results of GI tract were lacking in the evidence of granuloma and eosinophilic infiltrates. Results showed that patients in group II tended to have higher prevalence of involvement in nasal cavity and heart, and subsequently higher BVAS score and FFS. However, there were no differences between the two groups concerning the usage, dosage, and duration of GCs treatment at baseline. These data were shown in **Table 2**.

**Table 2:** Characteristics of EGPA patients with GI involvement (n=21).

	Biopsy: eosinophilia and/or granulomas in GI	No biopsy evidence of GI	P value
	(n=12)	(n=9)	
Demographic features			
Gender (Female (%))	5 (41.7%)	3 (33.3%)	0.697
Age at EGPA onset (yr)	39.3 ± 20.5	40.9 ± 11.7	0.833
Duration before diagnosis (m)	47.5 (15-156)	24 (7.5-42)	0.247
Patients received GCs at baseline	10 (83.3%)	5 (55.6%)	0.163
Baseline GCs dosage (Prednisone or equivalent dose, mg/d)	32.5 (18.8-46.3)	10.0 (7.5-47.5)	0.448
Baseline GCs duration(m)	1.7 ± 0.4	1.1 ± 0.5	0.405
Clinical manifestations			
Weight loss	8 (66.7%)	6 (66.7%)	1.000
Skin involvement	7 (58.3%)	5 (55.6%)	1.000
Nasal cavity involvement	7 (58.3%)	9 (100.0%)	***0.045***
Asthma	11 (91.7%)	5 (55.6%)	0.119
Renal involvement	2 (16.7%)	3 (33.3%)	0.611
Cardiac involvement	1 (8.3%)	5 (55.6%)	***0.046***
Peripheral neuropathy	7 (58.3%)	6 (66.7%)	1.000
CNS manifestation	1 (8.3%)	4 (44.4%)	0.119
Laboratory tests			
ANCA positivity Eosinophil count (10^9^/L) Eosinophil (%) ESR (mm/h) hs-CRP (mg/L) Serum IgE (kU/L)	3 (25.0%) 3.0 (3.0-9.0) 40.4 (24.8-44.0) 39.5 (5.0-58.0) 41.0 (16.5-115.5) 332.0 (162.0-422.0)	2 (25.0%) 3.0 (2.3-4.5) 35.6 (21.8-44.8) 70.5 (17.3-82.5) 98.0 (55.0-170.5) 501.0 (277.0-1421.0)	1.000 0.143 0.776 0.283 0.240 0.206
Scoring			
BAVS	17 (12-21)	30 (18-36)	***0.015***
FFS			
0 1 2 3	1 (8.3%) 8 (66.7%) 3 (25.0%) 0 (0.0%)	0 (0.0%) 3 (33.3%) 2 (22.2%) 4 (44.4%)	1.000 0.198 1.000 ***0.021***

when p value< 0.05, it is highlighted in bold.

All the GI group patients underwent abdominal imaging examinations (shown in **Table 3**). Among the 9 patients who had conducted abdominal computed tomography (CT) scan, 7 (77.8%) of them showed thickening of the gastrointestinal wall. The other positive findings by abdominal CT scan were ascites (22.2%, 2/9) and pelvic effusion (11.1%, 1/9), while 2 (22.2%) patients had negative findings. For the evaluation of mesenteric vessels, 3 patients completed vascular ultrasonography (US) and 2 patients underwent abdominal CT three-dimensional vascular reconstruction, among which 1 patient had superior mesenteric vein thrombosis. Meanwhile, five patients performed plain abdominal radiograph, among which 1 patient was observed to have GI perforation and one patient presented as ileus. In addition, among the 2 patients who completed PET/CT scan, one patient showed increased intestinal metabolism and the other one showed intestinal tympanites.

**Table 3:** Symptoms, affected site, treatment, and follow-up of EGPA patients with GI involvement.

	Age/sex	GI symptom	Affected site	Induction therapy	Follow-up
1	30/M	Abd. Pain, Abd. distention, diarrhea	Stomach, colon	MP+CTX	Remission
2	31/M	Abd. pain, nausea/vomiting	Stomach, duodenum, small intestine	MP+CTX	Remission
3	26/F	Abd. pain, diarrhea, nausea/vomiting	Stomach, duodenum	MP+CTX +CsA	Lost
4	52/M	Abd. pain	Duodenum	MP+CTX	Relapse
5	80/F	Poor appetite, Abd. distention, diarrhea	Stomach, duodenum, small intestine, colon, rectum	MP+CTX	Death^b^
6	65/M	Abd. pain	Stomach	MP+CTX	Relapse
7 8	27/M 70/F	Abd. pain Abd. Pain, diarrhea	Stomach, duodenum Rectum	MP+CTX MP+CTX	Remission Relapse
9	39/F	Abd. Pain, nausea/vomiting, GI bleeding, perforation	Stomach, duodenum	MP+CTX	Death^a^
10	63/M	Abd. Pain, diarrhea	Colon	MP	Relapse
11	64/M	Abd. Pain, GI bleeding	Colon	MP	Death^b^
12	24/M	Abd. Pain, acid regurgitation, vomiting, melena	Duodenum	Pred+CTX	Relapse
13	40/F	Abd. pain, diarrhea, melena, perforation	Stomach, duodenum	MP+CTX	Death^a^
14	37/M	Abd. pain	Duodenum	MP+CTX	Relapse
15	49/M	Abd. pain, ileus	Small intestine	MP+CTX	Remission
16	26/M	Abd. pain, nausea/vomiting, diarrhea, GI bleeding, perforation	Small intestine, colon, rectum	MP+CTX	Death^a^
17	54/F	Abd. pain	Uncertain	MP+CTX	Death^a^
18	34/M	Abd. pain, diarrhea, melena	Stomach	MP+ CTX+ CsA	Relapse
19	58/F	Acid regurgitation, heartburn	Stomach	Pred+CTX	Remission
20	58/F	Abd. pain, diarrhea	Stomach, duodenum, small intestine, Esophagus	Pred+CTX	Remission
21	29/M	Abd. pain	Small intestine, colon	MP+CTX	Remission

Abd, abdominal; CTX, cyclophosphamide; CsA, Cyclosporine A; F, female; M, male; MP, methylprednisolone; Pred, prednisone; Death^a^, patients died during hospitalization; Death^b^, patients died during follow-up.

In brief, according to the results of endoscopy, histopathology and abdominal imaging, stomach was the most common affected GI organ in EGPA patients with GI involvement, with 11 (52.4%) patients manifesting as gastric involvement, followed by duodenum (47.6%, 10/21), small intestine (33.3%, 7/21), colon (28.6%, 6/21), rectum (14.3%, 3/21) and esophagus (4.8%, 1/21) (**Figure 2B**).

### Potential risk factors for GI involvement in EGPA patients

According to univariate analysis, factors with two-sided p<0.10 were selected to explore potential risk factors of GI involvement in EGPA patients by multivariate logistic regression analysis (**Table 4**). In order to avoid the confounding effect of treatment at baseline, we adjusted the model for GCs treatment at baseline. We found that only weight loss (odds ratio, OR = 4.304, 95% CI 1.339–13.841) was the potential risk factor for GI involvement in EGPA patients.

**Table 4:** Multivariate analysis for 21 EGPA patients with GI involvement and 73 controls.

Variable	Odd Ratio	95% CI	P value
Weight loss Allergic rhinitis Peripheral neuropathy involvement Gender Age Disease period (month) Age at disease onset	***4.304*** 1.877 0.452 0.545 0.945 1.001 1.046	***1.339-13.841*** 0.635-5.551 0.136-1.510 0.211-1.975 0.784-1.209 0.983-1.015 0.815-1.257	***0.014*** 0.255 0.197 0.306 0.624 0.904 0.698

This model was adjusted for GCs use at baseline. OR, 95% CI and p value of independent risk factor (weight loss) were highlighted in bold.

### Treatment, follow-up, and outcomes of EGPA patients

The treatment strategies for all EGPA patients with or without GI involvement were summarized in **Table 3** and **Table 5**. Among patients in GI group, 4 (19.0%) patients received methylprednisolone pulse therapy (0.5-1.0g/day for 3-5 days), 15 (71.4%) patients received large-dose glucocorticoids (Prednisone 1-2 mg/kg/d or equivalent dose), and only 2 (9.5%) patients received medium- to low-dose glucocorticoids (Prednisone ≤ 0.5mg/kg/d or equivalent dose) for induction treatment. As for immunosuppressive agents, 20 (95.2%) patients in GI group received cyclophosphamide (CTX) as induction treatment, and only 1 patient with GI involvement did not receive CTX due to abnormal liver function. In addition to CTX, 2 (9.5%) patients in GI group also had Cyclosporine A (CsA) as induction treatment. Moreover, among the 3 patients with GI perforation, 2 patients accepted emergency surgery to fix the small intestine perforation, while the other patient’s family gave up surgery considering her general condition was too poor. A total of 17 (81.0%) patients in GI group achieved remission after induction treatment. However, 4 (19.0%) patients died during hospitalization. Among them, 3 patients died of GI perforation despite 2 of them accepted surgical intervention and 1 patient died of EGPA-related intracranial hemorrhages. During the follow-up period, 1 patient in GI group died of GI perforation at 3 months after discharge from hospital, and another patient died of severe pneumonia and septic shock at 32 months later.

**Table 5:** Induction treatment and outcomes of EGPA patients with and without GI involvement.

	GI group	Non- GI group	P value
	(n=21)	(n=73)	
Treatment, n (%)			
GCs MP pulse therapy CTX CsA IVIG AZA MTX MMF	21 (100%) 4 (19.0%) 20 (95.2%) 2 (9.5%) 0 (0%) 0 (0%) 0 (0%) 0 (0%)	73 (100%) 23 (31.5%) 61 (83.6%) 1 (1.4%) 6 (8.2%) 2 (2.7%) 4 (5.5%) 1 (1.4%)	- 0.266 0.285 0.124 0.332 1.00 0.572 0.285
Outcomes, cumulative rate (%)			
1-year relapse	19.2%	3.8%	***0.03***
3-year relapse	54.6%	13.1%	***<0.001***
1-year survival 3-year survival	75.2% 67.7%	100.0% 100.0%	***<0.0001*** ***<0.0001***

when p value< 0.05, it is highlighted in bold.

Additionally, the induction treatment strategies of patients in non-GI group were listed in **Table 5**. There was no significant difference between patients in GI group and non-GI group concerning induction treatment (**Table 5**). As for maintenance treatment strategy, low-dose GCs plus immunosuppressants, including CTX, methotrexate (MTX), azathioprine (AZA) and MMF were selected according to individual situations.

**Figure 3:**
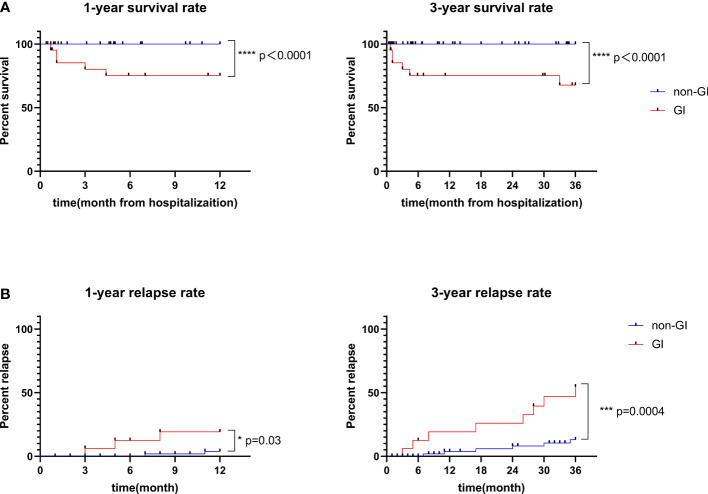
Survival rate **(A)** and relapse rate **(B)** of EGPA patients with or without GI involvement.

The follow-up data of all patients were also collected, with the median (IQR) follow-up period of 38 (14-75) months in GI group and 38 (28-94) months in non-GI group (P=0.746). Patients in GI group showed lower 1-year cumulative survival rate (75.2% vs. 100.0%, P <0.0001) and lower 3-year cumulative survival rate (67.7% vs. 100.0%, P<0.0001) compared with patients in non-GI group (**Figure 3A**). Furthermore, we also calculated the cumulative relapse rate (**Figure 3B**) of the two groups. Patients in GI group had higher 1-year cumulative relapse rate (19.2% vs. 3.8%, P=0.03) and higher 3-year cumulative relapse rate (54.6% vs. 13.1%, P<0.001) compared with patients in non-GI group. In addition, GI group had lower long-term remission rate compared with non-GI group [7 (33.3%) vs. 63 (86.3%), P<0.001].

## Discussion

To the best of our knowledge, this is the first study focusing on GI involvement in a relatively large cohort of EGPA patients. In this study, GI involvement occurred in 22.3% of EGPA patients at baseline, and EGPA patients with GI involvement had distinct clinical characteristics compared with EGPA patients without GI involvement. A Japanese study proposed that GI involvement is more likely to occur in elder EGPA patients (23), which was not observed in our study. CRP is an acknowledged marker of disease activity in vasculitis (15), and patients with GI involvement in this cohort had higher hs-CRP level and higher BVAS score than those without GI involvement, demonstrating that patients with GI involvement had higher disease activity compared with patients without GI involvement.

Similar to the results of previous studies (7, 10, 11), the most common symptoms in EGPA patients with GI involvement in our study were abdominal pain, followed by diarrhea, nausea and vomiting. In addition, several patients with GI involvement had untypical gastrointestinal symptoms, such as poor appetite, heartburn, and acid regurgitation, but subsequent radiograph findings or endoscopy confirmed the GI involvement of EGPA. Therefore, clinicians should also pay attention to mild GI symptoms in the evaluation of EGPA patients (7). Besides, weight loss of EGPA patients is a hint to reflect the severity of disease and an important item in the BAVS scoring system (13, 15, 16 ). Our results verified that weight loss was also a pivotal potential risk factor for GI involvement in EGPA, which indicated the importance of GI tract evaluation in EGPA patients with weight loss.

According to previous studies, EGPA related GI involvement could occur in any part of the digestive tract (23, 24) and the most common involved region was small intestine (25, 26). In our cohort, the most commonly involved region of the GI tract were stomach and duodenum, followed by small intestine and colon, while involvement of rectum and esophagus was relatively rare. Furthermore, the most common site of perforation was the small intestine, which was also reported by previous cases (25, 27, 28). Extra-vascular granuloma and eosinophilic infiltrates are the hallmarks of GI involvement in EGPA (3, 29, 30), which was detected in 80% of patients with GI involvement who completed histopathological in our cohort. However, non-specific acute or chronic inflammatory infiltrates were also found in systemic vasculitis (31), and patients without extra-vascular granuloma and eosinophilic infiltrates in the affected GI site could not rule out the diagnosis of GI involvement, since they had severe GI manifestations that couldn’t be explained by other mechanisms or achieved remission dramatically after treatment (18, 19). As shown in our cohort, among the patients in the GI group who did not undergo biopsy examination, five (87.7%) of them had severe GI involvement including GI perforation, GI bleeding and bowel obstruction. However, diagnosis of GI involvement could not be ruled out in this situation and comprehensive evaluation and timely management should be initiated to prevent the progression of the disease. As for the two patients lacking in the evidence of granuloma and eosinophilic infiltrates in GI tract, they achieved disease remission after addition or increasing dosage of GCs, which confirmed the diagnosis of GI involvement. The most common abnormality found by abdominal CT scan was the thickening of GI wall, which was suggestive of GI involvement but not diagnostic. Besides, plain abdominal radiograph had advantage in detecting GI perforation and ileus. As a result, imaging examinations are important but still have limitations to identify GI involvement, and clinical manifestations, endoscopy plus biopsy are also critical for the diagnosis of GI involvement in EGPA patients (32, 33).

As a risk factor for poor prognosis and a leading cause of death, patients with GI involvement are recommended to accept aggressive medical treatment and surgical intervention when indicated (34). GCs combined with CTX is indicated as the first-line treatment for induction of remission in severe disease, which could improve the prognosis of EGPA (21, 35–37). Consistently, all except only one EGPA patient with GI involvement accepted GCs plus CTX as induction treatment in our cohort, and 81.0% of them achieved remission after treatment. In addition, although GCs had a pivotal role in both the remission induction and the maintenance therapy of EGPA patients with GI involvement, there are still challenges due to the GI side effect of GCs (35, 38). For one thing, high dose GCs can contribute to the elevated risk of GI perforation. Several cases have reported that EGPA patients with GI involvement developed sudden GI perforation after high dose GCs therapy (25, 39) and GCs associated perforation was likely to happen in patients with potential GI inflammatory lesion (40). For another, the prevalence of GI bleeding and ulcers as adverse effects of GCs are also positively correlated with the increasing dose of GCs (41, 42), especially when GCs were used in combination with nonsteroidal anti-inflammatory drugs (43). Therefore, GCs sparing strategy was important to lower the dosage of GCs (28). Moreover, a large proportion of EGPA patients need a long-term GCs therapy to control disease related manifestations (44, 45), thus the GI side effects caused by long-term use of GCs should provoke attention, especially for EGPA patients with GI involvement.

Several previous studies have reported higher mortality rate in systemic vasculitis patients with GI involvement (31, 46, 47), and some researchers have also identified GI involvement as one of the main causes of death or signs of poor prognosis in EGPA patients (23, 48). A retrospective study of EGPA patients in Japan also mentioned that patients with GI or cardiac involvement were more likely to experience disease relapse (19), although their results showed no significant differences in mortality between patients with or without GI involvement. In our study, the cumulative survival rate of EGPA patients with GI involvement during a median follow-up time of 38 months reached 84.4%. Consistent with the higher FFS scores indicating of poorer prognosis, patients with GI involvement had higher relapse rate and higher mortality, but lower long-term remission rate compared with patients in non-GI group, indicating the maintenance treatment and management strategies should be carefully chosen for this subgroup of patients (21, 35, 38, 49).

Although the incidence of GI bleeding (14.3%) and perforation (14.3%) was relatively low in our cohort, patients with these events always had poor prognosis. In our cohort, all the 3 patients died of GI perforation despite proper medical treatment and surgical intervention. As shown in previous EGPA cases, although there were also reports of successful treatment after perforation (28, 50, 51), intestinal perforation usually led to high mortality (25–27, 52). Therefore, early use of intensified immunosuppressors such as CTX or B cell depletion strategy besides glucocorticoids (53) were recommended to avoid life-threatening events for patients with severe GI involvement (51). The occurrence of GI bleeding are also precursors of poor prognosis; therefore clinicians need to pay full attention to this condition and give sufficient intervention at early stage.

The major limitation of this study lies in the fact that this is a retrospective study from a single center, thus bias may exist in the process of data collection. Future prospective multicenter studies are warranted to establish more findings.

In conclusion, GI involvement is common in EGPA patients in our cohort, and patients with GI involvement have distinct characteristics from those without GI involvement, including higher hs-CRP level, higher BVAS and FFS scores. Moreover, weight loss is identified as a potential risk factor for GI involvement in EGPA patients in our cohort. Compared with EGPA patients without GI involvement, EGPA patients with GI involvement showed lower cumulative survival rate, lower long-term remission rate and higher relapse rate.

## Data availability statement

The original contributions presented in the study are included in the article/**Supplementary Material**. Further inquiries can be directed to the corresponding authors.

## Ethics statement

The studies involving human participants were reviewed and approved by Ethics Committee of Peking Union Medical College Hospital. The ethics committee waived the requirement of written informed consent for participation.

## Author contributions

RL and YC collected and analyzed the data and wrote the manuscript. SZ and LP collected and analyzed the data and revised the manuscript. WZ and YZ designed the study, re-checked the diagnosis of all patients and revised the manuscript. YF and JZ designed the study, analyzed the data and wrote the manuscript. All authors contributed to the article and approved the submitted version.
