# Supplementary material for: Clinical characteristics and long-term outcome of patients with gastrointestinal involvement in eosinophilic granulomatosis with polyangiitis
Source: Front Immunol. 2023 Jan 12;13:1099722. doi: 10.3389/fimmu.2022.1099722 (PMC9879136; doi:10.3389/fimmu.2022.1099722)
Supplement: Supplementary file 1 [file Table_1.docx]

Table S1. Characteristics of EGPA patients with GI involvement (n=21).

| patients | GI manifestations as initial symptoms | GI endoscopy examination | GCs treatment at baseline (duration) | Treatment response |
| --- | --- | --- | --- | --- |
| 1 | Yes | Yes, no biopsy | No | GI perforation, emergency surgery |
| 2 | Yes | No | No | Small bowel obstruction improved significantly after GC treatment |
| 3 | Yes | No | No | GI bleeding improved significantly after GC treatment |
| 4 | No | Yes, no biopsy | GC 10mg/day (3 months) | GI perforation, emergency surgery |
| 5 | No | No | GC 45mg/day (1 month) | Abdominal pain improved after increasing dosage of GC |
| 6 | No | Yes, no biopsy | GC 10mg/day (2 weeks) | Abdominal pain and diarrhea improved significantly after increasing dosage of GC |
| 7 | No | Biopsy, but no evidence of eosinophils (endoscopy findings are consistent with eosinophilic enteritis) | No | Abdominal pain, diarrhea and vomiting improved significantly after GC treatment |
| 8 | No | Biopsy, but no evidence of eosinophils | GC 50mg/day (3 weeks) | Abdominal pain improved after increasing dosage of GC |
| 9 | No | Yes, no biopsy (endoscopy findings are consistent with EGPA related ulcers) | GC 5mg/day (1 week) | GI bleeding and perforation |
| 10 | No | Yes, biopsy: eosinophils infiltration | GC 15mg/day (3 months) | Abdominal pain improved after increasing dosage of GC |
| 11 | Yes | Yes, biopsy: eosinophils infiltration | GC 45mg/day (6 weeks) | Abdominal pain, distention and diarrhea improved significantly after increasing dosage of GC |
| 12 | No | Yes, biopsy: eosinophils infiltration | GC 5mg/day (2 months) | Abdominal pain and diarrhea improved significantly after increasing dosage of GC |
| 13 | Yes | Yes, biopsy: eosinophils infiltration | GC 20mg/day (4 months) | Abdominal pain improved significantly after increasing dosage of GC |
| 14 | No | Yes, biopsy: eosinophils infiltration | GC 50mg/day (3 weeks) | Abdominal distention and diarrhea improved significantly after increasing dosage of GC |
| 15 | No | Yes, biopsy: eosinophils infiltration | GC 60mg/day (2 weeks) | Abdominal pain improved significantly after GC pulse |
| 16 | No | Yes, biopsy: eosinophils infiltration | No | Abdominal pain and diarrhea improved significantly after GC treatment |
| 17 | Yes | Yes, biopsy: granulomas | GC 20mg/day (3 months) | Abdominal distention and diarrhea improved significantly after increasing dosage of GC |
| 18 | No | Yes, biopsy: eosinophils infiltration | GC 30mg/day (1 month) | Abdominal pain, acid regurgitation, vomiting and melena improved significantly after increasing dosage of GC |
| 19 | No | Yes, biopsy: eosinophils infiltration | GC 35mg/day (2 weeks) | Acid regurgitation improved significantly after increasing dosage of GC |
| 20 | No | Yes, biopsy: eosinophils infiltration | No | Abdominal pain and diarrhea improved after GC treatment |
| 21 | No | Yes, biopsy: eosinophils infiltration | GC 40mg/day (2 weeks) | Abdominal pain improved significantly after increasing dosage of GC |
